# Cardiorespiratory fitness data from 18,189 participants who underwent treadmill cardiopulmonary exercise testing in a Brazilian population

**DOI:** 10.1371/journal.pone.0209897

**Published:** 2019-01-09

**Authors:** Joao Manoel Rossi Neto, Antonio Sergio Tebexreni, Alexandre Novakoski Ferreira Alves, Paola Emanuela Poggio Smanio, Floriana Bertini de Abreu, Mauricio Cruz Thomazi, Priscilla Ayumi Nishio, Ivana Antelmi Cuninghant

**Affiliations:** Fleury Medicina e Saúde, Sao Paulo–SP, Brazil; Sao Paulo State University - UNESP, BRAZIL

## Abstract

**Purpose:**

Cardiorespiratory fitness is inversely associated with a high risk of cardiovascular disease, all-cause mortality, and mortality attributable to various cancers. It is often estimated indirectly using mathematical formulas for estimating oxygen uptake. Cardiopulmonary exercise testing, especially oxygen uptake, represents the “gold standard” for assessing exercise capacity. The purpose of this report was to develop reference standards for exercise capacity by establishing cardiorespiratory fitness values derived from cardiopulmonary exercise testing in a Brazilian population. We focused on oxygen uptake standards and compared the maximal oxygen uptake [mLO_2_·kg^-1^·min^-1^] values with those in the existing literature.

**Methods:**

A database was constructed using reports from cardiopulmonary exercise testing performed at Fleury laboratory. The final cohort included 18,189 individuals considered to be free of structural heart disease. Percentiles of maximal oxygen uptake for men and women were determined for six age groups between 7 and 84 years. We compared the values with existing reference data from patients from Norway and the United States.

**Results:**

There were significant differences in maximal oxygen uptake between sexes and across the age groups. In our cohort, the 50th percentile maximal oxygen uptake values for men and women decreased from 44.7 and 36.3 mLO_2_·kg^-1^·min^-1^ to 28.4 and 22.3 mLO_2_·kg^-1^·min^-1^ for patients aged 20–29 years to patients aged 60–69 years, respectively. For each age group, both Norwegian men and women had greater cardiorespiratory fitness than cohorts in the United States and Brazil.

**Conclusion:**

To our knowledge, our analysis represents the largest reference data for cardiorespiratory fitness based on treadmill cardiopulmonary exercise testing. Our findings provide reference values of maximal oxygen uptake measurements from treadmill tests in Brazilian populations that are more accurate than previous standard values based on workload-derived estimations. This data may also add information to the global data used for the interpretation of cardiorespiratory fitness.

## Introduction

Oxygen uptake (VO_2_max) is considered to be the most important parameter associated with an individual's physical conditioning, and it is an objective and independent prognostic indicator for cardiovascular disease [1]. Cardiorespiratory fitness (CRF) is inversely associated with a high risk of cardiovascular disease, all-cause mortality, and mortality attributable to various cancers [2]. Improvements in CRF are associated with reduced mortality risk, and small increases in CRF (e.g., 1–2 METs) are associated with considerably lower (10–30%) adverse cardiovascular event rates [2,3].

CRF is often estimated indirectly using mathematical formulas to estimate VO_2_ uptake. Cardiopulmonary exercise testing (CPX), especially VO_2_ uptake, is the most widely used and reliable test for assessing exercise capacity [2]. In addition, it is very important to have accurate reference values for CRF owing to the relevance of CRF in estimating health risks, since CRF varies according to age, sex, and population [2].

In Brazil, the most widely cited reference data are derived from the Cooper Clinic, which uses estimated CRF values calculated using treadmill speed and grade [4]. In 2003, the ATS/ACCP Statement on Cardiopulmonary Exercise Testing stated that the criteria for the classification of functional class (level of fitness) should be based on CPX results [5]. In 2013, the American Heart Association affirmed the need to develop a registry that directly measured normative values of VO_2_ uptake [6]. The limited data available that directly measures VO_2_ uptake using treadmill CPX makes it difficult to compare CRF between countries. Recently, the publication of data from the United States [7] and Norway [8,9] has helped identify the normative values for VO_2_ uptake during treadmill exercise in different regions of the world. In 2016, a scientific statement from the American Heart Association, which collected the United States data, recommended that, ideally, all adults should have CRF estimated by a maximal test using CPX, and if CPX is not feasible, a non-exercise algorithm should be used to estimate CRF to enhance risk prediction [2].

The purpose of this report was to develop reference standards for exercise capacity by establishing CRF values derived from CPX in a Brazilian population, and we used the United States publication as a guideline because of its large sample size, similar age distribution, and comparability with the Norway data. This report will focus on VO_2_max standards from treadmill testing, and we will compare the results with the existing literature from Norway and the United States [4,7–9].

## Methods

### Participants

We analyzed the data collected from consecutive individuals who underwent CPX between January 1, 2000, and May 31, 2016, in the Fleury Laboratory units. The following variables were available in this report: indications for the test, age, weight, height, medications, whether the VO_2_ uptake was considered maximum or peak, the value of VO_2_ uptake (mL.kg-1.min-1 and mL.min-1), if the resting electrocardiogram traces were normal or altered (ischemia, bundle branch block, second and third AV block, atrial fibrillation, left ventricular hypertrophy, and pre-excitation syndrome), or if the test result was considered abnormal (ischemic or suggestive of ischemia) or normal. A database was constructed using these variables. The inclusion criteria were: checkup or aerobic evaluation as the indication, VO_2_max, a normal electrocardiogram, normal test results, and no medication that could influence the VO_2_ uptake.

The exclusion criteria were: VO_2_ peak, altered electrocardiogram results (see inclusion criteria), abnormal test results (see inclusion criteria), or medications (beta blockers, medications for chronic obstructive pulmonary disease, or antiarrhythmics) that could influence VO_2_ uptake.

With these criteria, we were able to obtain the VO_2_max in a population considered to be free of structural heart disease and compare the results with the data from the United States and Norway.

Our population was mostly from the city of Sao Paulo (a megalopolis with many immigrants, cultures, and ethnicities), but as the tests were conducted by a private entity, the participants had a higher socioeconomic status and may not have represented the entire Brazilian population.

### VO_2_max

We used the criteria by Howley [10] and Balady [11] to define the VO_2_max criteria that was maintained for the entire cohort. VO_2_max was defined by two or more of the following criteria: 1) respiratory exchange ratio (RER) >1.10, 2) at least 95% of the age-predicted maximal heart rate [220 − age (in y)], 3) plateau in the VO_2_ uptake curve despite increasing the exercise intensity until exhaustion (≤2.1 mL.kg-1.min-1 to the next level), or 4) clinical volitional exhaustion (maximal voluntary effort according to the Borg scale that ranges from very, very easy = 1 to exhaustion = 10). Peak VO_2_ was defined as not meeting the criteria for VO_2_max. Samples were obtained breath by breath and averaged over 30-second time frames. If a plateau was not reached, the highest VO_2_max during a 30-second stage was used.

All institutional units used the Vmax Encore (SensorMedics, Norma Linda, CA) device. Flow calibration was performed by a 3-l syringe, and gas analyzers were calibrated using two standard gases (gas 1: 16% O_2_, 4% CO_2_; gas 2: 26% O_2_, 0.0% CO_2_) according to the recommended manufacturer instructions prior to each use.

### Treadmill protocol

The ramp treadmill protocol was used for all tests and was based on the patient's previous aerobic condition, being individualized with a 2-minute warm-up phase starting as low as 4.0 km/h and increasing at increments of 1.0 km/h, up to the tolerance limit of the subject. All tests started at a grade of 0%, and the grade was increased up to 20% (the objective was to have most tests fall within the 8 to 12-minute range). The average maximal velocity and grade during the test protocol were 12.0 km/h (range 4–20 km/h) and 4.5% (range 0–20%), respectively. The CPX were carried out according to the recommended standards provided in the recently published guidelines [12,13].

### Ethics statement

The study was approved by the review board/ethics committee of Fleury Institute (CAAE: 63362116.1.0000.5474) and complied with the Declaration of Helsinki. The Fleury Institute review board/ethics committee considered informed consent unnecessary owing to the characteristics of this study (retrospective database analysis).

### Statistical analyses

Descriptive data are presented as mean ± standard deviation (SD), whereas categorical data are reported as frequencies (percentages). We used an analysis of variance to compare differences in VO_2_max values between the sexes and across age groups. To determine differences via analysis of variance, the Tukey test was applied for post-hoc analysis if significance was observed. The Student's t-test was used to compare the mean VO_2_max results of our study (according to sex/age range) and these values in the existing literature [7–9]. SPSS statistical software, version 22.0 (IBM Corp., Armonk, NY), was used for all analyses. All tests with a significance of P<0.05 were considered statistically significant.

## Results

The initial cohort included 24,929 tests. We excluded 5,262 tests because they were considered to be peak VO_2_, 704 because they had electrocardiogram changes, 812 because of medication use that could influence the VO_2_max results, and 235 with incomplete data. The final cohort included 18,189 tests, 12,555 men and 5,634 women ranging in age from 7–84 years. Overall, the VO_2_max was 39.9±8.6 mL.kg-1.min-1 (range 11.0–75.7 mL.kg-1.min-1). We included only three individuals older than 80 years, and the VO_2_max for all these individuals revealed a mean of 24.0±5.4 mL/kg/min. In the age group ≤12 years, the mean age was 11.4±1.2 and 11.2±0.7 and the mean VO_2_max was 46.3±9.5 and 44.7±7.5 for boys (n = 22) and girls (n = 13), respectively. In the age group of 70–79 years, we had 65 tests, 49 men and 16 women with a mean VO_2_max of 33.7±7.1 mL/kg/min and 26.5±5.7 mL/kg/min, respectively. Descriptive characteristics of the cohort by sex and age groups are listed in Table 1. VO_2_max during CPX are also presented in Table 1 according to the previous definition.

**Table 1:** Descriptive characteristics of the Fleury cohort*.

	Age group (y)*						
	13–19	20–29	30–39	40–49	50–69	60–69	ALL
***Men***	***n = 381***	***n = 1201***	***n = 4427***	***n = 4383***	***n = 1728***	***n = 362***	***n = 12552***
**Age. y**	16.5±1.8	25.7±2.8	35.0±2.8	44.0±2.8	53.4±2.7	63.3±2.7	40.2±10.2
**Height (cm)**	177.2±7.8	177.9±6.8	177.9±6.7	177.3±6.6	176.4±6.2	174.8±6.4	177.3±6.8
**Weight (kg)**	73.5±14.8	80.2±11.8	82.8±11.4	82.8±11.6	82.3±11.1	81.1±11.5	82.1±11.8
**BMI**	23.3±3.9	25.3±3.1	26.1±3.0	26.3±3.1	26.5±3.2	26.5±3.2	26.1±3.2
**VO**_**2**_**max**	48.9±7.9	45.0±7.5	43.5±7.9	41.6±7.8	38.6±7.9	33.7±7.1	42. ±8.3
**aVO**_**2**_**max**	3.5±0.6	3.6±0.6	3.6±0.6	3.4±0.6	3.1±0.5	2.7±0.5	3.4±0.6
***Women***	***n = 110***	***n = 732***	***n = 2028***	***n = 1985***	***n = 624***	***n = 128***	***n = 5634***
**Age. y**	16.6±1.9	25.9±2.6	34.9±2.8	43.9±2.7	53.4±2.7	63.5±2.7	39.3±9.7
**Height (cm)**	164.7±6.6	164.8±6.3	164.4±6.0	163.5±5.9	162.8±5.9	160.8±5.4	163.8±6.1
**Weight (kg)**	62.2±11.5	61.0±9.1	62.1±9.8	62.5±9.2	62.9±9.9	62.6±9.8	62.2±9.6
**BMI**	22.9±3.8	22.4±3.0	23.0±3.3	23.4±3.1	23.7±3.3	24.2±3.6	23.2±3.2
**VO**_**2**_**max**	37.4±7.7	36.9±6.6	36.0±7.0	34.7±7.1	31.4±6.5	26.5±5.7	35.0±7.3
**aVO**_**2**_**max**	2.3±0.5	2.2±0.4	2.2±0.4	2.1±0.4	1.9±0.4	1.6±0.3	1.5±0.3

BMI: body mass index (kg/m^2^); VO_2_max: relative maximal oxygen uptake (mLO_2_·kg^-1^·min^-1^); aVO_2_max = absolute VO_2_max (mLO_2_·min^-1^)

*Data are presented as mean±SD.

**Fig 1:**
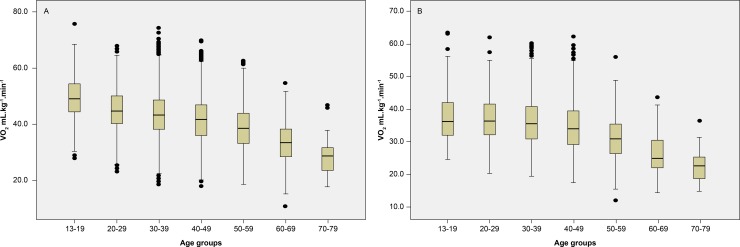
Maximal oxygen uptake. Boxplot of measured maximal oxygen uptake (VO_2_max [mLO2·kg-1·min-1]) in the Fleury cohort obtained from men (A) and women (B) performing treadmill exercise tests across age groups. Error bars indicate SD.

Fig 1 shows that the VO_2_max was lower in each ascending age group.

For both men and women, the percentile values for each age group from the Fleury data, the previously published data from the Cooper Clinic, [4] and the Fitness Registry and the Importance of Exercise National Database (FRIEND) [6] are shown in Table 2.

**Table 2:** Age- and sex-specific percentiles for CRF in FRIEND [7], Fleury, and previously published data from the Cooper Clinic [4] (VO_2_max [mLO_2_·kg^-1^·min^-1^] measured using treadmill CPX tests).

	Percentile						
Age group (y)	5^th^	10^th^	25^th^	50^th^	75^th^	90^th^	95^th^
**Men (FRIEND)**							
20–29	29.0	32.1	40.1	48.0	55.2	61.8	66.3
30–39	27.2	30.2	35.9	42.4	49.2	56.5	59.8
40–49	24.2	26.8	31.9	37.8	45.0	52.1	55.6
50–59	20.9	22.8	27.1	32.6	39.7	45.6	50.7
60–69	17.4	19.8	23.7	28.2	34.5	40.3	43.0
70–79	16.3	17.1	20.4	24.4	30.4	36.6	39.7
**Men (Cooper)**							
20–29	31.8	34.7	39.0	43.9	48.5	54.0	55.5
30–39	31.2	33.8	37.8	42.4	47.0	51.7	54.1
40–49	29.4	32.3	35.9	40.1	44.9	49.6	52.5
50–59	26.9	29.4	32.8	37.1	41.8	46.8	49.0
60–69	23.6	25.6	29.5	33.8	38.3	42.7	45.7
70–79	20.8	23.0	26.9	30.9	35.2	39.5	43.9
**Men (Fleury)**							
20–29	33.2	35.4	40.3	44.7	50.1	55.3	57.8
30–39	30.6	33.2	38.1	43.3	48.7	53.6	56.5
40–49	28.9	31.4	36.1	41.7	46.9	51.9	54.7
50–59	25.6	28.2	33.0	38.6	43.9	49.2	51.9
60–69	22.6	24.5	28.2	33.4	38.2	42.8	45.8
**Women (FRIEND)**							
20–29	21.7	23.9	30.5	37.6	44.7	51.3	56.0
30–39	19.0	20.9	25.3	30.2	36.1	41.4	45.9
40–49	17.0	18.8	22.1	26.7	32.4	38.4	41.7
50–59	16.0	17.3	19.9	23.4	27.6	32.0	35.9
60–69	13.4	14.6	17.2	20.0	23.8	27.0	29.4
70–79	13.1	13.6	15.6	18.3	20.8	23.1	24.1
**Women (Cooper)**							
20–29	27.6	29.5	33.0	37.8	42.4	46.8	49.6
30–39	25.9	28.0	32.0	36.7	41.0	45.3	47.4
40–49	25.1	26.6	30.2	34.5	38.6	43.1	45.3
50–59	23.0	24.6	28.0	31.4	35.2	38.8	41.0
60–69	21.8	23.0	25.1	28.8	32.3	35.9	37.8
70–79	19.6	21.5	24.2	27.6	29.8	32.5	37.2
**Women (Fleury)**							
20–29	26.4	28.6	32.2	36.4	41.6	45.7	47.9
30–39	25.2	27.2	30.9	35.6	40.9	45.3	47.8
40–49	24.0	26.0	29.3	34.1	39.5	44.3	46.8
50–59	21.7	23.6	26.4	30.9	35.4	41.0	43.3
60–69	18.7	20.2	22.1	25.0	30.5	34.5	38.0

CRF: Cardiorespiratory fitness; CPX: cardiopulmonary exercise testing; FRIEND: Fitness Registry and the Importance of Exercise National Database; Cooper: Cooper Clinic; VO_2_max: maximal oxygen uptake (mLO_2_·kg^-1^·min^-1^). All patients are considered to be free of known cardiovascular disease. The FRIEND CRF data were measured with CPX. The Cooper Clinic data reported were predicted from the Balke test time or work rate. The Fleury data were measured with CPX

We could not perform formal statistical comparisons owing to the unavailability of individual participant data from the Cooper Clinic cohort. Therefore, the data presented in Table 2 are for observation purposes only. Compared with the Cooper Clinic data, the 50^th^ percentile data for men in the Fleury registry were higher in the 20-, 30-, 40-, and 50-year-old age groups and lower in the 60- and 70-year-old age groups. The same comparison made with the FRIEND registry showed that only the 20-year-old age group had data below the 50^th^ percentile. Comparing the Fleury cohort with the Cooper clinic data, the 50^th^ percentile values were lower for the women in the 20- and 70-year-old age groups, and when the Fleury data were compared to the FRIEND registry data, 50^th^ percentile data were lower only in the 20-year-old group, with the rest of the age groups being higher.

Table 3 shows the comparison by sex of the four studies that used CPX data with the same age group distribution [7–9]. For each age group, Norwegian [8,9] men and women had greater cardiorespiratory fitness than those in the United States [7] and Brazil.

**Table 3:** Age- and sex-specific comparison of mean reference values for CRF in the Fleury data and previously published values (VO_2_max [mLO_2_·kg^-1^·min^-1^] measured using treadmill CPX tests).

	Age group (y)					
Sex	20–29	30–39	40–49	50–59	60–69	70–79
**Male**						
FRIEND [7]	47.6±11.3 (n = 513)	43.0±9.9 (n = 963)	38.8±9.6 (n = 1327)	33.8±9.1 (n = 1078)	29.4±7.9 (n = 593)	25.8±7.1 (n = 137)
Loe [8]	54.4±8.4 (n = 199)	49.1±7.5 (n = 324)	47.2±7.7 (n = 536)	42.6±7.4 (n = 466)	39.2±6.7 (n = 300)	35.3±6.5 (n = 76)
Edvardsen [9]	48.9±9.6 (n = 38)	46.2±8.5 (n = 73)	42.7±9.3 (n = 91)	36.8±6.6 (n = 88)	32.4±6.4 (n = 81)	30.1±4.8 (n = 23)
Fleury	45.0±7.5 (n = 1201)	43.5±7.9 (n = 4427)	41.6±7.8 (n = 4383)	38.6±7.9 (n = 1728)	33.7±7.1 (n = 362)	28.7±6.7 (n = 48)
**Female**						
FRIEND [7]	37.6±10.2 (n = 410)	30.9±8.0 (n = 608)	27.9±7.7 (n = 843)	24.2±6.1 (n = 805)	20.7±5.0 (n = 408)	18.3±3.6 (n = 98)
Loe [8]	43.0±7.7 (n = 215)	40.0±6.8 (n = 359)	38.4±6.9 (n = 493)	34.4±5.7 (n = 428)	31.1±5.1 (n = 240)	28.3±5.2 (n = 53)
Edvardsen [9]	40.3±7.1 (n = 37)	37.6±7.5 (n = 63)	33.0±6.4 (n = 86)	30.4±5.1 (n = 79)	28.7±6.6 (n = 59)	23.5±4.1 (n = 41)
Fleury	36.9±6.6 (n = 732)	36.0±7.0 (n = 2028)	34.7±7.1 (n = 1985)	31.4±6.5 (n = 624)	26.5±5.7 (n = 128)	23.4±5.9 (n = 14)

CPX: cardiopulmonary exercise testing; CRF: Cardiorespiratory fitness; FRIEND: Fitness Registry and the Importance of Exercise National Database; VO_2_max: maximal oxygen uptake (mLO_2_·kg^-1^·min^-1^)

## Discussion

The current analysis represents, to our knowledge, the largest study of reference data on treadmill cardiorespiratory fitness using data obtained from CPX. In Brazil, the largest existing reference studies evaluated a distribution of age groups different from those observed in this study, such as in Herdy's first report of 3,992 exams [14] and the second report of 9,250 exams [15].

In conjunction with the literature, our findings show a reduction in cardiorespiratory fitness with increasing age, regardless of sex. The differences in CRF between the sexes appear to be greater in the early stages of life and begin to decline in older individuals, with a more pronounced difference in the elderly. It is interesting to note that when we use the absolute values (mLO_2_·min-1), this decline becomes more linear. Because of the clear importance of CRF for patients’ health and prognosis, the quantification of reference values on a global scale to provide region-specific data is of paramount importance. Currently, owing to the limited data available, we can only speculate that these differences in the age-related decline could be due to the level of previous physical conditioning, hereditary and genetic predisposition, socioeconomic status, nutritional level, sports culture, emotional stress, and other factors. The principal similarity between the studies was that the vast majority of participants were apparently healthy.

In our institution, the most widely used data for CRF referrals are from the Cooper Clinic. These were estimated from the workload on the maximal stress test or by the total test time using the Balke protocol [4]. As indicated in Table 3, the results by sex and age range in the Fleury record are higher in the 20-, 30-, 40- and 50-year-old age groups and slightly lower in the 60- and 70-year-old age groups compared to those from the Cooper Clinic.

We cannot explain the differences between our results and the Cooper Clinic data. However, as mentioned by the FRIEND Registry [7], this may be related to the Balke protocol, “which can cause local fatigue of calf muscles and potentially an early test termination. This would result in a lower predicted VO_2_max” [7]. In fact, the Balke protocol presented characteristics that compromised the VO_2_max measurement, especially when the test exceeded 15 minutes, leading to early fatigue due to velocity and increased incline, especially in individuals with reduced physical conditioning. The FRIEND Registry argues that “furthermore, the equations used to estimate VO_2_max from treadmill speed and grade were only validated for submaximal steady-state exercise; thus, these equations are known to over-predict VO_2_ at higher levels of exercise. In addition, although handrail use is discouraged, if not well regulated, it will result in the ability to tolerate higher work rates on a treadmill exercise test at a lower oxygen cost, which could lead to overestimation of VO_2_max” [7]. In any case, the results obtained from CPX are different from those derived from mathematical equations based on velocity and grade.

When comparing the bicycle exercise mode with the cycle ergometer, the VO_2_max is typically 10–20% smaller when performing maximum CPX on a cycle ergometer [16,17]. The main problems that existed with the old equations were that they were derived from small samples and were extrapolated from the bicycle to the treadmill. With the publication of several databases with a large population with the direct measurement of VO_2_max, a great interest was developed in the development of equations to estimate VO_2_max. Recently, the FRIEND equation predicted the VO_2_max with an overall error >4 times lower than the error associated with the traditional American College of Sports Medicine equations (5.1±18.3% vs. 21.4±24.9%, respectively) [18,19]. Souza and Silva developed a bicycle and treadmill equation derived from the FRIEND data and compared this equation with those that previously existed, and they found that the VO_2_max values derived using the former equation were significantly closer to the actual VO_2_max values than that calculated using the older equations. Several factors influence the CPX results, and we have demonstrated differences between the largest databases in our study. Therefore, the new VO_2_max estimation equations may not accurately measure physical fitness. Thus, we believe that direct measurement of VO_2_max should be the method of choice for assessing an individual's CRF.

The strength of this study is that it provides reference data for VO_2_max measured from CPX in a large Brazilian population. These results should preferably be used for patients with a good socioeconomic status being evaluated for a physical fitness assessment. Our results are, perhaps, inadequate for the general population of Brazil, since it is probable that the level of physical conditioning, nutritional status, and socioeconomic level is lower in the general population of Brazil. It should be noted that we tried to rule out any preexisting structural disease, results, or drugs that could influence the VO_2_max result. Nevertheless, the sample size was large, and it provides more appropriate reference values in relation to the VO_2_max estimation equations for laboratories that include CPX as part of the maximal exercise test measurements.

Some limitations should be considered that are common to all studies that use retrospective data. Patients with known cardiovascular disease, with electrocardiographic alterations before and after the test, and those taking medications known to interfere with VO_2_max were excluded from the study. However, the term “considered to be free of structural heart disease” would not be appropriate for the entire study population because some individuals may have risk factors for cardiovascular disease (diabetes, obesity, etc.). Although all tests were performed to measure functional capacity, the choice of treadmill protocols was specific to each contributing institutional unit. While the sample size was large, the numbers of participants varied among the age groups, with the greatest representation in the 30- and 40-year-old age groups, and a lesser representation of those over 70 years old (approximately 0.4% of the total sample). Our results suggest that future studies should seek greater representation from the younger and older age groups. All the tests were carried out in the Fleury laboratory units in the city of São Paulo, a megalopolis with more than 12 million people, but it was not possible to determine the patients’ geographical distribution. Finally, because we did not have access to the Cooper data, statistical tests were not performed, and we were limited to performing only observational comparisons, similar to the study from the FRIEND group [7].

The search for normative values for CRF is a worthy pursuit, and there is a clear need to define cutoff points for what is “fit” versus “unfit” by sex and age groups in relation to morbidity and mortality outcomes. Previous studies using the Cooper Clinic data have defined “unfit” as the bottom 20% and the “fit” as the upper 80% of the VO_2_max distribution [20,21]. Unfortunately, we do not have morbidity or mortality data showing the relationship between CRF and all-cause/cardiovascular disease mortality in Brazil, so we usually extrapolated data from the United States.

The current analysis represents, to our knowledge, the largest reference standard for cardiorespiratory fitness using data obtained from CPX. These values should provide interpretations of the VO_2_max measurements from treadmill tests in a Brazilian population that are more accurate than previous standards that were based on workload-derived estimations of VO_2_max. This new VO_2_max data may also add more information to the global data used for the interpretation of cardiorespiratory fitness.
